# Cloning and Characterization of 5′ Flanking Regulatory Sequences of *AhLEC1B* Gene from *Arachis Hypogaea* L.

**DOI:** 10.1371/journal.pone.0139213

**Published:** 2015-10-01

**Authors:** Guiying Tang, Pingli Xu, Wei Liu, Zhanji Liu, Lei Shan

**Affiliations:** 1 Bio-Tech Research Centre, Shandong Academy of Agricultural Sciences / Shandong Provincial Key Laboratory of Crop Genetic Improvement, Ecology and Physiology, Jinan, 250100, China; 2 College of Agriculture, Shandong University, Jinan, 250100, China; McGill University, CANADA

## Abstract

LEAFY COTYLEDON1 (LEC1) is a B subunit of Nuclear Factor Y (NF-YB) transcription factor that mainly accumulates during embryo development. We cloned the 5′ flanking regulatory sequence of *AhLEC1B* gene, a homolog of *Arabidopsis LEC1*, and analyzed its regulatory elements using online software. To identify the crucial regulatory region, we generated a series of GUS expression frameworks driven by different length promoters with 5′ terminal and/or 3′ terminal deletion. We further characterized the GUS expression patterns in the transgenic *Arabidopsis* lines. Our results show that both the 65bp proximal promoter region and the 52bp 5′ UTR of *AhLEC1B* contain the key motifs required for the essential promoting activity. Moreover, *AhLEC1B* is preferentially expressed in the embryo and is co-regulated by binding of its upstream genes with both positive and negative corresponding *cis*-regulatory elements.

## Introduction

NF-Y (Nuclear Factor Y) transcription factor is ubiquitous in eukaryotic organisms. The three subunits of NF-Y, NF-YA, NF-YB, and NF-YC, play an important role in regulating the expression of multiple genes (both positively and negatively) by recognizing and binding to the CCAAT promoter sequence [1, 2]. In the *Arabidopsis* genome, there are 36 NF-Y subunits, including 10 NF-YA, 13 NF-YB and 13 NF-YC. These subunits are differentially expressed in a tissue- or organ-specific pattern, or in the distinctive profile of developmental stages, and participate in regulating of many genes in a wide range of biological processes [3–5].

The NF-Y transcription factor genes such as *LEAFY COTYLEDON1* (*LEC1* or *NF-YB9*) and *LEC1-LIKE* (*L1L* or *NF-YB6*)–first identified in *Arabidopsis*–are genes related to embryonic development. *AtLEC1* and *AtL1L* mRNA accumulate in different spatial and temporal patterns. Higher levels of *AtLEC1* mRNA are present in the early-stage embryo at the proembryo stage, globular stage, transition stage, heart stage, torpedo stage, and curled cotyledon stage than in the late maturation embryo, but is not detectable in leaves, stems, roots, and flowers [6], while *AtL1L* mRNA levels are higher in seeds than in vegetative tissues. *AtL1L* RNA levels peak at a later stage of embryogenesis (mainly from the torpedo stage to the bent-cotyledon stage) as compared with *LEC1* levels. Warpeha et al. (2007) [7] showed *NF-YB6* and *NF-YB9* expression in the 6-d-old etiolated seedlings of *Arabidopsis*. Siefers et al. (2009) [3] identified 36 nuclear factor transcription subunits that can combine to govern tissue-specific expression patterns of flowering time, embryo maturation, meristem development, etc. in *Arabidopsis*. The *turnip* (*tnp*) mutant represents a gain-of-function mutant of Arabidopsis LEC1. In *tnp* mutant, the elements required for the repression of *LEC1* in vegetative tissue are deleted in the distal upstream promoter region causing a higher constitutive expression of *LEC1* [8].

Here, we analyze the phylogenetic relationship among the peanut transcription factors *AhLEC1A*, *AhLEC1B*, and the *Arabidopsis* NF-YB transcription factors. We also cloned the 5′ flanking regulatory sequence of the *AhLEC1B* gene and analyzed the *cis*-regulatory elements existing in this region by computational analyses. We further constructed a set of GUS expression frameworks driven by different length promoters with 5′ terminal and 3′ terminal deletion to identify the crucial regulatory regions and characterize the GUS expression patterns in their transgenic *Arabidopsis* lines.

## Materials and Methods

### Plant materials and growth conditions

Peanut (*Arachis hypogaea* L.) cv. ‘Luhua 14’ seeds were grown in the experimental field of Shandong Academy of Agricultural Sciences. Seeds at different developmental stages were collected at 10~70 days after pegging (DAP) and kept in -80°C refrigerator for isolation of total RNA and construction of a cDNA library.

### Cloning of 5' flanking region of *AhLEC1*

Peanut genomic DNA was isolated from Luhua 14 leaves using CTAB method [9]. For each DNA library construction, 2.5μg genomic DNA was digested with four blunt-end restriction enzyme *DraI*, *EcoRV*, *PvuII*, and *StuI* respectively. The digested samples were purified with phenol and chloroform; and then 4μl digested DNA was connected with the BD GenomeWalker adaptor (Table 1) provided by BD GenomeWalker Universal Kit (Clontech, USA), resulting in the library containing digestions by *DraI*, *EcoRV*, *PvuII*, and *StuI* (LD, LE, LP, and LS). Based on the sequence of *AhLEC1B* genomic DNA (S1 Fig), two nested gene-specific primers (GSP), LEC1BGSP1-2 and LEC1BGSP2-2 (Table 1), were designed. The first round of PCR reaction was done as per the manufacturer’s instructions in a 25μl reaction system using an AP1 (Table 1) provided by Kit and LEC1BGSP1-2 as 5' terminal and 3' terminus primer, and 1μl DNA of each library as template. The nested PCR reaction was also performed using the same volume and conditions with primers AP2 (Table 1) and LEC1BGSP2-2, and 1μl of the 10-fold diluted primary PCR products as template. The specific PCR fragments from the second round reaction were isolated and inserted into the vector pEASY-T3. The recombinants harboring the target gene were validated by *EcoRI* digestion and two-way sequencing using ABI3730 model DNA sequencer.

**Table 1:** The primers used in this study.

Serial No.	Primer name	Sequences	Purposes
1	BD GenomeWalker adaptor	5' GTAATACGACTCACTATAGGGCACGCGTGGTCGACGGCCCG GGCTGGT 3'	No.1~5 used for the amplification of 5′ flanking sequence of *AhLEC1B* genome DNA
2	LEC1BGSP1-2	5' CCTTGTTCCCATGTAAAACCATGAAAGCA 3'	
3	LEC1BGSP2-2	5' AGGTAAAGCAGCCGCTAATCTAGTTAGT 3'	
4	AP1	5' GTAATACGACTCACTATAGGGC 3'	
5	AP2	5' ACTATAGGGCACGCGTGGT 3'	
6	GeneRacer RNA Oligo	5' CGACUGGAGCACGAGGACACUGACAUGGACUGAAGGAG UAGAAA 3'	No.6~10 used for the localization of the transcriptional start site of *AhLEC1B* gene
7	TSS LEC1BGSP1-1	5' TCTTTTGCGTCGTCGGAGATTTTAGC 3'	
8	TSS LEC1BGSP2	same as LEC1BGSP2-2	
9	5' GeneRacer Primer	5' CGACTGGAGCACGAGGACACTGA 3'	
10	5' Nested Primer	5' GGACACTGACATGGACTGAAGGAGTA 3'	
11	BF1	5′ AAGCTTTCGTGAATAAAGGAACAC 3′	No.11~17 used for the deletion analysis of *AhLEC1B* promoter
12	BF2	5′ AAGCTTACTCTATGATATTCCGAAGG 3′	
13	BF3	5′ AAGCTTCCTCGGTTGCATCGCCCT 3′	
14	BF4	5′ AAGCTTGCATTGCTTGCAGCTCTTTG 3′	
15	BF5	5′ AAGCTTGTTACTCCGTTTCTTCATAC 3′	
16	BR1	5′ CCATGGGTAAAGCAGCCGCCAATCTA 3′	
17	BR2	5′ CCATGGCTCGCCCTTCGGAATATCAT 3′	

### Localization of transcriptional start site

High-quality total RNA was isolated from Luhua14 mixed seeds at different developmental stages (ranging from 10 to 70 DAP) using the improved CTAB method [10]. According to the GeneRacer^TM^ Kit's recommendation, 5μg total RNA was dephosphorylated by Calf Alkaline Phosphatase (CIAP or CIP), and the full-length mRNA among them was removed using the 5' Cap structure, which was then ligated to the RNA adaptor (GeneRacer RNA Oligo, Table 1). The ds-cDNA was synthesized based on the manufacturer’s instruction using the above decapped, full-length mRNA with RNA Oligo as template, and oligo dT provided by SuperScript^TM^ III RT kit as a primer. The ds-cDNA was cloned into vector pCR4-TOPO to establish the full-length cDNA library.

For amplifying the transcription start site (TSS) of the target gene, two 3′ terminus gene-specific primers for each gene, TSS LEC1BGSP1-1 and TSS LEC1BGSP2 (Table 1), were designed, for use in the nested PCR reaction. The 5' terminus general primers for two rounds of PCR were 5' GeneRacer^TM^ Primer and 5' Nested Primer (Table 1). According to the recommended system of BD Advantage™ 2 PCR Kit, the primary PCR was performed as per the following conditions: 94°C denatured for 2 min, and 5 cycles of 94°C for 30 sec and 72°C for 30sec, and then 5 cycles of 94°C for 30 sec and 70°C for 30 sec, and 20 cycles of 94°C for 30 sec, 63°C for 30sec and 68°C for 30sec, and finally extension for 10 min at 68°C. The nested PCR was performed using a 50-fold dilution of the primary PCR product as template. The PCR condition were: denaturation at 94°C for 2 min; 35 cycles of 94°C for 30 sec, 65°C for 30 sec and 68°C for 10 sec; and finally 68°C for 10 min.

The nested PCR products were collected and sequenced by ABI3730 model DNA sequencer.

### Computational *cis-*regulatory motif analysis of the promoter of *AhLEC1B* gene

Two different online software PLACE (http://www.dna.affrc.go.jp/PLACE/) and PlantCARE (http://bioinformatics.psb.ugebp.be/webtools/plantcare/html/) were used to predict the *cis*-regulatory elements in the 5' flanking region of *AhLEC1B* gene, including the 5' untranslated region (5' UTR) and the upstream regulatory region.

### Constructs of GUS expressing system, *Arabidopsis* transformation, and GUS staining

The different length promoters with 5' or 3' terminal deletion were obtained by PCR. All primers are listed in Table 1. BR1 and BR2 are reverse primers localized in 5' UTR of *AhLEC1B*. BF1-BF5 are the forward primers situated in the different sites of the *AhLEC1B* promoter (Table 1). For cloning purposes, a *HindIII* site (AAGCTT) and a *NcoI* site (CCATGG) was added to the 5' border and 3' border of each fragment by PCR amplification with an appropriately designed oligonucleotide. The six fragment-deleted promoters replacing the CaMV 35S promoter were cloned into pCAMBIA3301 digested with *HindIII* and *NcoI*.

The binary vectors constructed above were transferred into *Agrobacterium tumefaciens* strain GV3101 and then transformed into *Arabidopsis* Col-0 plants using the floral dip method [11]. Seeds were harvested and stored at room temperature. For screening, seeds were sterilized in 95% (v/v) ethanol for 1 min and 0.1% (v/v) HgCl for 20 min, followed by several washes with sterile water. Herbicide-resistant plants were selected by incubating plants for 14d on MS [12] basal medium supplemented with10 mg/L Basta.

GUS staining was performed using a standard protocol [13]. The roots and leaves at the 4-leaf stage, stems at the bolting stage, flowers, and seeds of 6–10 days after pollination in transgenic T_2_ lines were incubated with the staining buffer (0.1% TritonX-100 and 2mM 5-bromo-4-chloro-3-indolyl-β-D-glucuronide (X-Gluc), cyclohexyl ammonium salt in 100mM sodium phosphate buffer, pH7.0) at 37°C overnight or 24h and then decolorized with 70% ethanol. The analyses were performed using at least six independent transgenic lines for analysis.

## Results

### Phylogenetic analysis of AhLEC1A and AhLEC1B

**Fig 1:**
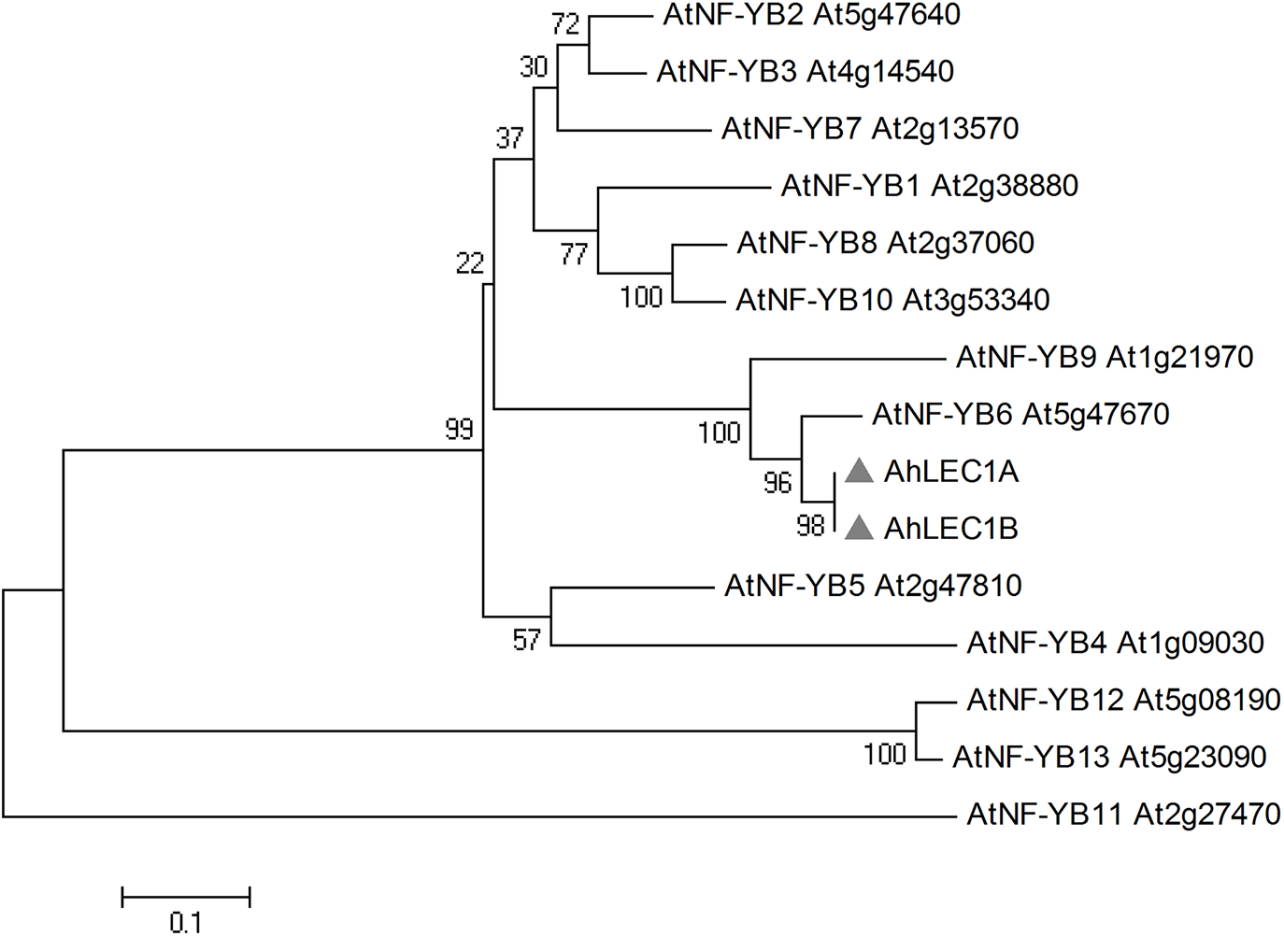
Phylogenetic tree for peanut AhLEC1A and AhLEC1B, and the *Arabidopsis* NF-YB family.

In the *Arabidopsis* genome 13 *NF-YB* genes with distinctive expression patterns were found [3–5]. To predict the evolutionary relationship of AhLEC1A and AhLEC1B, a sequence comparison of AhLEC1A, AhLEC1B, and Arabidopsis NF-YB transcription factors was performed using MAGE 4.0. AhLEC1A, AhLEC1B, *Arabidopsis* NF-YB6 (L1L) and NF-YB9 (LEC1) have higher sequence similarity and group together (Fig 1). AhLEC1A and AhLEC1B share 95% sequence identity and diverge at only 12 amino acid sites. However, the expression profile of *AhLEC1A* was substantially different from that of *AhLEC1B*. *AhLEC1A* is expressed specifically in seeds during different developmental stages while *AhLEC1B* mRNA accumulates at higher levels in seeds as compared with roots, stems, rosettes, and flowers [14].

### Cloning and sequence analysis of 5' flanking region of *AhLEC1B* and localization of TSS

**Fig 2:**
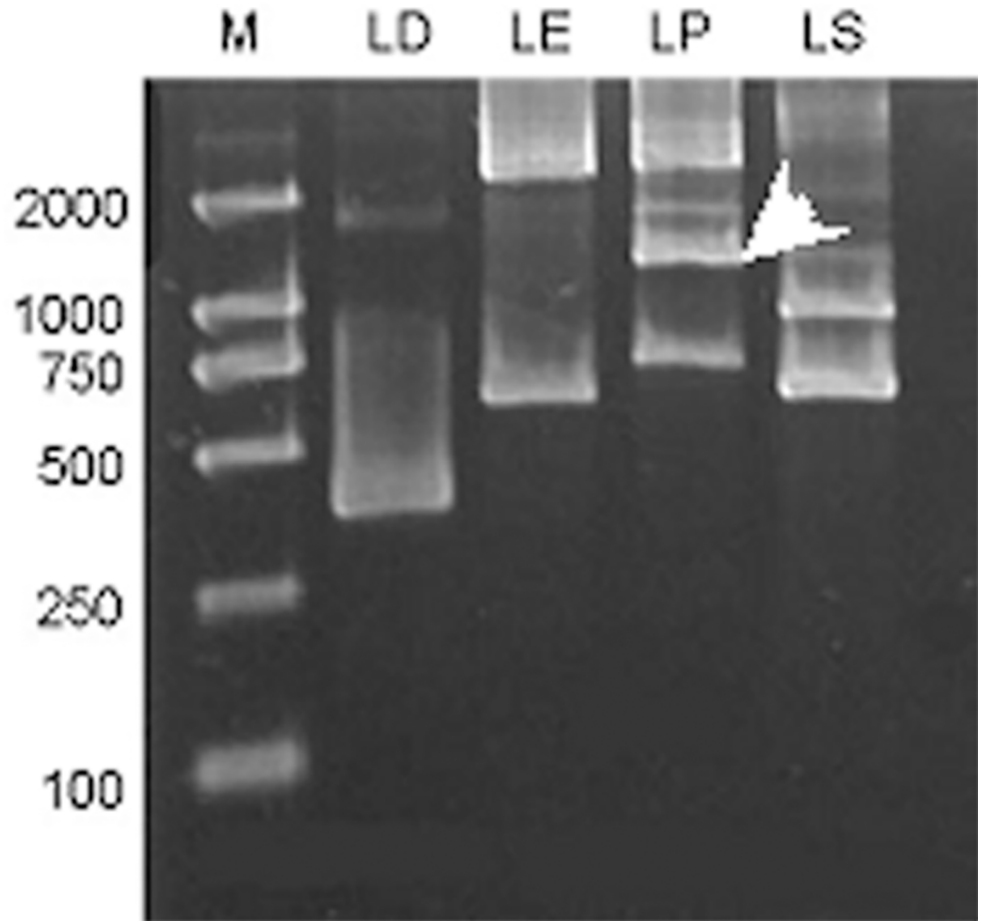
The second round of PCR amplification products of 5′ flanking regulation regions of peanut *AhLEC1B* gene by chromosome walking. The arrow indicates the target band.

To investigate the major regulatory regions or elements of *AhLEC1B*, we isolated the promoter using chromosomal walking. As a result, the 5' flanking fragment of 1289 bp in length including the promoter region (1235bp) and 5'UTR sequences (54bp) was obtained from the peanut DNA library LP (Fig 2)

**Fig 3:**
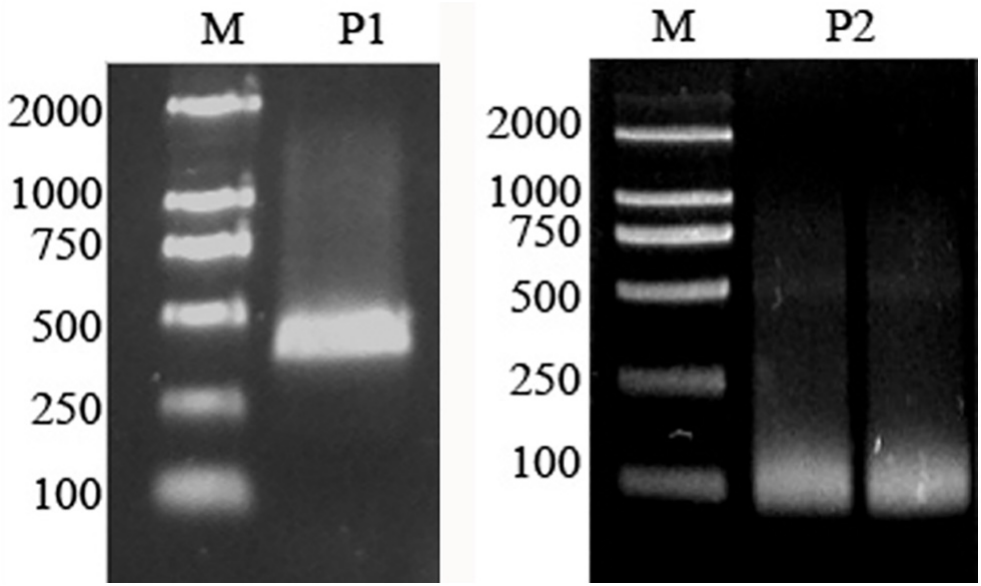
Localization of transcription start sites of the peanut *AhLEC1B* gene using 5′ RACE. P1 –Product of the first round PCR; P2 –Product of the second round PCR

**Fig 4:**
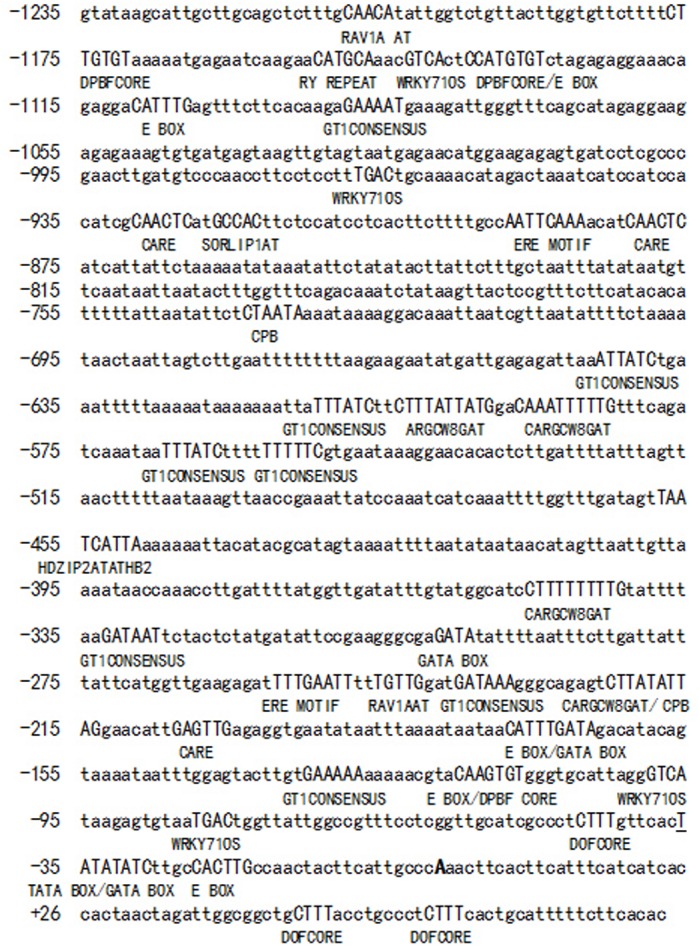
The sequence of 5′ flanking regulation region of peanut *AhLEC1B* gene and some major elements harbored in this region. The bold capital letter “A” represents the transcription start site (TSS), and other capital letters show different regulatory elements.

Based on the cDNA sequence of *AhLEC1B*, we further amplified the 5'UTR of the gene from the full-length cDNA library of Luhua14 developing seeds using nested 5' RACE. As a result, we obtained PCR products of about 400bp and 60bp (Fig 3). The transcription of *AhLEC1B* gene starts at the first ‘A’ within the sequence of CCAAACT. This sequence is located 83 bp upstream to the translation start codon ATG, and is consistent with the general feature in most eukaryotes (Fig 4).

### *Cis*-elements prediction of *AhLEC1B* promoter

To predict *cis*-regulatory elements in 5' flanking fragment of *AhLEC1B*, we submitted the 1318 bp sequences containing 1235 bp promoter region and 83 bp 5'UTR to PLACE and PlantCARE online to detect *cis*-regulatory elements.

The putative TATA box (TATATAT) in the core region of promoter was located –36 from TSS. The other *cis*-regulatory elements were classified into two groups (Fig 4). The first group contains the multiple-copied elements, such as E BOX (CANNTG, 5 copies), CARGCW8GAT (CWWWWWWWWG, 4 copies), SEF4 MOTIF (RTTTTTR, 7 copies) and the DOF CORE (AAAG, 17 copies). All of these elements exist in the regulatory regions of many genes that are preferentially expressed in the seed or embryo [15–18]. Moreover, the CACTFT (YACT, 22 copies), TAAAG MOTIF (5 copies), ROOT MOTIF (ATATT, 15 copies), OSE2 ROOT NODULE (CTCTT, 8copies) and POLLEN1 LELAT52 (AGAAA, 7 copies) are expressed in leaf, root and flower, respectively [19–22]. Some motifs required for light regulation (twelve copies of GATA BOX and ten copies of GT1 CONSENSUS) are dispersed in the promoter region of *AhLEC1B* [23, 24]. Four copies of TGAC core sequences (WRKY71OS) were also scattered in the promoter region. Zhang et al. (2004) [25] found that the TGAC core motif could bind with rice WRKY71 transcriptional repressor to participate in the regulation of the gibberellin signaling pathway. The second group of *cis*-regulatory elements included a large number of elements with lower copies (less than three copies) or a single copy. These include several phytohormone-regulated elements such as CPB Sequence (TATTAG, cytokinin response), ERE Motif (AWTTCAAA, ethylene-induced transcription), GARE Motif (TAACAGA, Gibberellin-responsive element) [26–28], and some elements (TGACGT Sequence and PROLAMIN BOX) related to gene expression levels [29, 30], and some tissue- or organ-preferential regulatory elements DPBF CORE (ACACNNG, associated with embryo- or seed-preferential expression), and RAV1A AT (CAACA) which expresses in relatively higher level in rosette leaves and roots, and etc (Fig 4) [31, 32]. A copy of RY REPEAT sequence (CATGCA) was present in the upstream region of *AhLEC1B* promoter. RY REPEAT sequence is present in the promoters of many genes regulating seed development [33] and is also found in the promoter and intron regions of *AtLEC1*. Other specific *cis*-regulatory elements, such as CELL CYCLE BOX (CACGAAAA) and HEXAMER MOTIF (ACGTCA) were present in *AhLEC1B* promoter. The CELL CYCLE BOX (CACGAAAA) is involved in cell-cycle-specific activation of transcription [34] while HEXAMER MOTIF (ACGTCA) functions in the regulation of replication-dependent expression of the histone H3 gene [35, 36]. They all exist mainly by the style of a single copy in the promoter region of this gene.

### GUS expression driven by *AhLEC1B* promoter fragments

**Fig 5:**
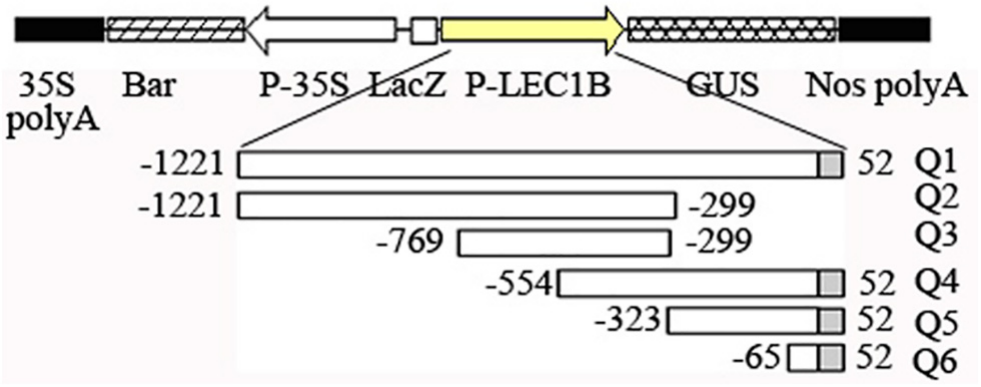
The constructs of GUS expression driven by *AhLEC1B* promoter and schematic representation of the different length promoters with 5′ or 3′ terminal deletion. Q1-Q6 indicates the different promoters with 5′ or 3′ terminal deletion. The white and gray rectangles show the upstream promoter region from TSS and 5′ UTR region respectively.

**Fig 6:**
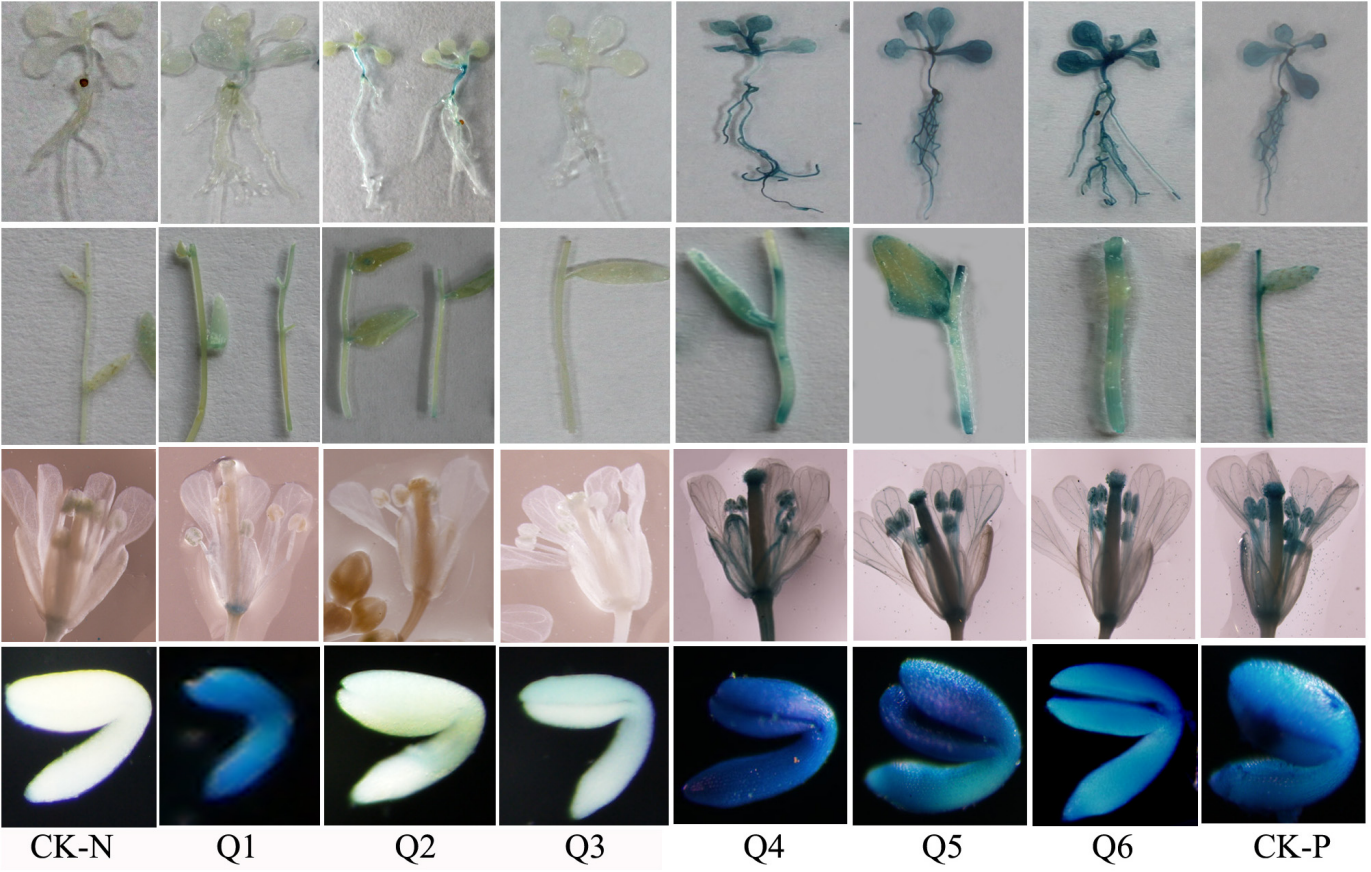
Effects of *AhLEC1B* promoter deletions on the expression profile of *GUS* gene in transgenic Arabidopsis lines. Q1-Q6 indicate the GUS expression patterns in different transgenic Arabidopsis lines containing 5′ or 3′ terminal deletion promoters, and the CK-N and CK-P showed the GUS expression profiles in non-transformed negative control and in positive control harboring 35S:GUS constructs, respectively.

To identify the crucial regulatory regions that are essential for gene expression, we generated a series of constructs containing different length *AhLEC1B* promoter with 5' terminal deletion and 52bp 5' UTR or 3' terminal deletion fused with GUS reporter gene (Fig 5). All constructs were introduced into the *Arabidopsis* genome by *Agrobacterium*-mediated transformation. The resulting transgenic T_2_ lines containing a single copy homologous gene were screened for use in GUS histochemical staining studies. The results of staining in diverse tissues or organs showed that the longest fragment (Q1, 1281bp) containing 1229bp promoter region and 52bp 5' UTR, mainly regulates the GUS expression in the developing embryo. Moreover, three fragments (Q4, Q5, and Q6) with a 5' terminal deletion could drive the GUS expression in all tissues detected (Fig 6). However, the promoter fragment (Q2 and Q3) with 351bp deletion from 3' terminus lost the promoter function that had crucial activity responsive elements (Table 2). The shortest fragment (Q6, 118bp) including 66bp promoter region and 52bp 5' UTR contains the main elements that control the constitutive expression of the downstream gene (Fig 6).

**Table 2:** Major elements in 5′UTR and 300bp promoter region.

Elements	Sequence^a^	Location^b^ ^,^ ^c^	Putative Function
ACGT Sequence	ACGT	-120(+,-)	ACGT sequence (from -155 to -152) required for etiolation-induced expression of erd1 (early responsive to dehydration) in Arabidopsis [37].
ARR1AT	NGATT	-257(+), 36(+)	"ARR1-binding element" found in Arabidopsis; Required for transcriptional activation in response to cytokinin [38].
CACTFT PPCA1	YACT	-36(+), -21(+), -137(+), -10(+), -154(-), -139(-), -113(-), -88(-), 9(+), 28(+), 66(+)	Tetranucleotide (CACT) is a key component of Mem1 (mesophyll expression module 1, which direct mesophyll-specific expression of gene) [19].
CARE element	CAACTC	-203(-)	CAREs, CAACTC regulatory elements, are required for GA-inducible expression of hydrolase genes in the germinating seeds [39].
CARGCW8GAT	CWWWWWWWWG	-220(+,-)	A variant of CArG motif with a longer A/T-rich core is a preferential binding site for the transcriptional regulator AGL15 that accumulates during embryo development [17].
CCAAT BOX1	CCAAT	-72(-), 38(-)	Common sequence found in the 5'-non-coding regions of eukaryotic genes, which involved in increasing the promoter activity [40].
CPB Sequence	TATTAG	-216(+)	The sequence is critical for Cytokinin-enhanced Protein Binding in vitro [27].
CURE CORE	GTAC	-138(+,-), -118(+,-)	Copper-response element, also involved in oxygen-response of some genes [41].
DOF CORE	AAAG	-43(-), 49(-), 62(-)	Core site is required for binding of Dof proteins, which may be associated with the plant-specific pathway for carbon metabolism in maize [42].
DPBF CORE	ACACNNG	-115(-)	The binding core sequence of bZIP transcription factor DPBF-1 and 2 (Dc3 promoter-binding factor-1 and 2); Involved in embryo-specific expression, and responding to ABA [31].
E2F CONSENSUS	WTTSSCSS	-72(+)	E2F consensus sequence of all different E2F-DP-binding motifs that were involved in cell cycle regulation, DNA replication, and chromatin dynamics [43].
E BOX	CANNTG	-170(+,-), -115(+,-), -21(+,-)	The *cis*-elements in the promoter regions of most genes encoding the storage protein [18].
ERE Motif	AWTTCAAA	-253(-)	The ethylene responsive element mediate ethylene-induced activity of transcription [28].
GATA BOX	GATA	-297(+), -235(+), -165(+), -29(-)	Required for high level, light regulated, and tissue specific expression [23].
GT1 CONSENSUS	GRWAAW	-235(+), -130(+), 50(-), 73(-)	Consensus GT-1 binding site in the promoter regions of many light-regulated genes [24].
GTGA Motif	GTGA	-193(+), -132(+)	"GTGA motif" found in the promoter of the tobacco late pollen gene g10 and the tomato gene lat56, required for the gene expression in pollen [44]
I BOX CORE	GATAA	-235(+)	Conserved sequence upstream of light-regulated genes of both monocots and dicot.
POLLEN1 LELAT52	AGAAA	-285(-), 75(-)	One of two co-dependent regulatory elements (AGAAA and TCCACCATA) responsible for pollen specific activation of gene [21].
RAV1A AT	CAACA	-243(-)	Binding consensus sequence of Arabidopsis transcription factor RAV1, which expresses in relatively higher level in rosette leaves and roots [32].
ROOT MOTIF	ATATT	-294(+), -217(+), -190(-)	Motif found both in promoters of rolD, which expresses strongly in roots [20].
SEF4 MOTIF	RTTTTTR	-248(+)	Binding with SEF4, one of soybean embryo factor (SEF) [15].
SORLIP1 AT	GCCAC	-23(+)	One of "Sequences Over-Represented in Light-Induced Promoters (SORLIPs) in Arabidopsis; Involved in phyA-regulated gene expression [45].
TAAAG Motif	TAAAG	-233(+), 49(-)	TAAAG motif controls guard cell-specific gene expression [46].
WRKY71 OS	TGAC	-81(+), -96(-)	A core of TGAC-containing W-box; Binding site of rice WRKY71, a transcriptional repressor of the gibberellin signaling pathway or the regulation of the pathogenesis-related genes [25].

^a^N = G/A/C/T; R = A/G; S = C/G; W = A/T; Y = T/C

^b^The symbol ‘+’ or ‘-’ in the bracket represents the DNA strand in which the element is situated.

^c^The positive number indicates the location of element in 5′UTR, while the negative represents that in promoter.

## Discussion

*Arabidopsis LEC1* and *L1L* genes regulate embryogenesis, but they have distinct function during embryo development [6, 47]. *LEC1* expression in the embryos peaks at the early stage of seed development and declines thereafter, up to the green premature seed stage [6, 48]. The loss–of–function mutation in *LEC1* results in desiccation intolerance of embryos and defective in the production of storage proteins and lipids. However, as compared with *LEC1* levels, the *L1L* mRNA levels peak at the later stage of embryogenesis. The suppression of *L1L* in RNAi transgenic lines results in abnormal embryos and the embryo lethal phenotype [47], but its mutants *l1l-1* and *l1l-2* have no apparent altered phenotypes during seed development [49]. *AhLEC1A* and *AhLEC1B* from peanut are homologous genes of *Arabidopsis NF-YB6 (L1L)* and *NF-YB9 (LEC1)* and have differential expression patterns in vegetative tissues. Our RT-PCR data shows that *AhLEC1B* mRNA, as similar as *AtL1L* does, accumulates at a higher level in seeds but at a lower level in vegetative tissues [14]. Thus, our expression data and phylogenetic analysis together shows that *AhLEC1B* is an ortholog of *AtL1L*.

In this study, we cloned and analyzed the 5′ flanking regulatory sequence of *AhLEC1B*, and found that *GUS* gene, driven by the whole-length Q1 construct, preferentially expressed in embryos of the transgenic *Arabidopsis*. On the other hand, the transgenic lines with 452bp- 1156bp deletion constructs of Q1 from 5′ terminal showed higher GUS expression in roots, rosettes, stems, flowers, and seeds. Previous studies showed that the upstream region of *AtLEC1* promoter contains elements that repress its function in vegetative tissues [8]. Moreover, the seed-specific expression of the *AtLEC1* gene is controlled by combinatorial properties of negative and positive *cis*-regulatory elements in its promoter [8]. *PICKLE* (*PKL*)–a putative chromatin-remodeling factor–forms part of a NuRD histone deacetylase complex, which as a negative regulator of *AtLEC1* expression, represses embryonic identity and contributes to the transition from embryonic to postembryonic development in vegetative tissues [50, 51]. We hypothesize that the expression of *AhLEC1B* gene has a similar regulatory mode in peanut. The VP1/ABI3-LIKE (VAL) B3 proteins (as another repressor) in *Arabidopsis*, specific binding to the canonical sequence of Sph/RY *cis*-elemnets (CATGCA), are required for repression of the LEC1/B3 transcription factor network during gemination and vegetative development [33, 52]. Our results showed that an RY REPEAT element (CATGCA) localized at -1149bp of the *AhLEC1B* promoter region from TSS may be the binding site for VAL. The binding of VAL to the RY REPEAT element probably inhibits *AhLEC1B* expression in vegetative tissues. Moreover, the distal region of the *AhLEC1B* promoter consists of several other negative regulatory elements such as WRKY71OS (a transcriptional repressor of the gibberellin signaling pathway) and SRE (sugar-repressive element), which may be associated with upstream genes to decline its expression in particular way. Many elements required for the expression in embryo or endosperm, such as E BOX, CARGCW8GAT, and DPBF CORE, and so on, disperse in the Q1 construct. The E BOX elements are concentrated in the region from -250 to -50 in the promoters of some genes involved in fatty acid biosynthesis, triacylglycerol synthesis, and reserve including *SeFAD2*, *Cs-ACP1* and *Cs-4PAD*, *acyl-CoA-diacylglycerol acyltransferase* (At2g19450), *phosphatidylcholine*: *diacylglycerol acyltransferase* (At3g44830), several oil-body *oleoresins* (At3g01570, At3g18570, At3g27660, At5g40420, and At5g51210), and two *caleosins* (At4g26740 and At5g55240) [18]. The non-canonical CArG motif—CARGCW8GAT, which is an AGL15 (AT5G13790) transcription factor (TF) binding site is present in many endosperm-specific TF gene promoters [17, 53]. AGL15 might act upstream of the chalazal endosperm-specific TF genes and functions in activating at least one chalazal endosperm gene regulatory network [54].

The 300bp proximal region and 52bp 5′ UTR of the *AhLEC1B* promoter have crucial regulatory elements that are required for its basic activity and function. Deletion of these regulatory elements causes loss of reporter expression in Q2 transgenic lines (Fig 6). In this region, with the exception of TATA BOX, many tissue- or organ-specific elements, including phytohormone-responsive elements, light-regulated elements, elements associated with biotic stress and abiotic stress response, etc., were found (Table 2). Furthermore, the 5′-end deletion analysis of Q1 construct indicated that the 65bp promoter fragment with the 52bp 5′ UTR, where TATA BOX, CACTFTPPCA1, DOF CORE, GATA BOX, SORLIP1AT, and the like exist, could satisfy its basic driving function. The promoter also drives the GUS activity in a manner similar to that of the CaMV 35S promoter in all detected tissues. In general, plant promoters have a distal region (upstream activation sequence) and a proximal region (core region of the promoter) located at about 30-40bp upstream of TSS. In our study, we found that *AhLEC1B* promoter harbored within -65~+52bp region has those crucial elements such as DOF CORE and GATA BOX and the like. Morton et al. (2014) [55] found that ROEs (regions of enrichment) of transcription factor binding site (TFBS) in the proximal promoter region within 40 nucleotides from the TSS are present either in Narrow Peak promoters or in those of Broad with Peak, where these crucial elements helpfully determine the profiles and levels of gene expression.

In conclusion, the *AhLEC1B* gene–with transcripts preferentially in the embryo–is co-regulated by the binding of upstream genes and the corresponding *cis*-regulatory elements in its promoter. The promoter elements may negatively and positively regulate the gene, and its 65bp promoter region plus 52bp 5′ UTR contain the key motifs required for the essential promoter activity.

## Supporting Information

S1 Fig The sequence of *AhLEC1B* genomic DNA. The sequences underlined indicated the exon1 and exon2, and the italics showed the 5′UTR sequence. The start and stop codon were shown using the bold letters.
(TIF)
