# Supplementary figures and images for: Cloning and Characterization of 5′ Flanking Regulatory Sequences of AhLEC1B Gene from Arachis Hypogaea L
Source: PLoS One. 2015 Oct 1;10(10):e0139213. doi: 10.1371/journal.pone.0139213 (PMC4591277; doi:10.1371/journal.pone.0139213)

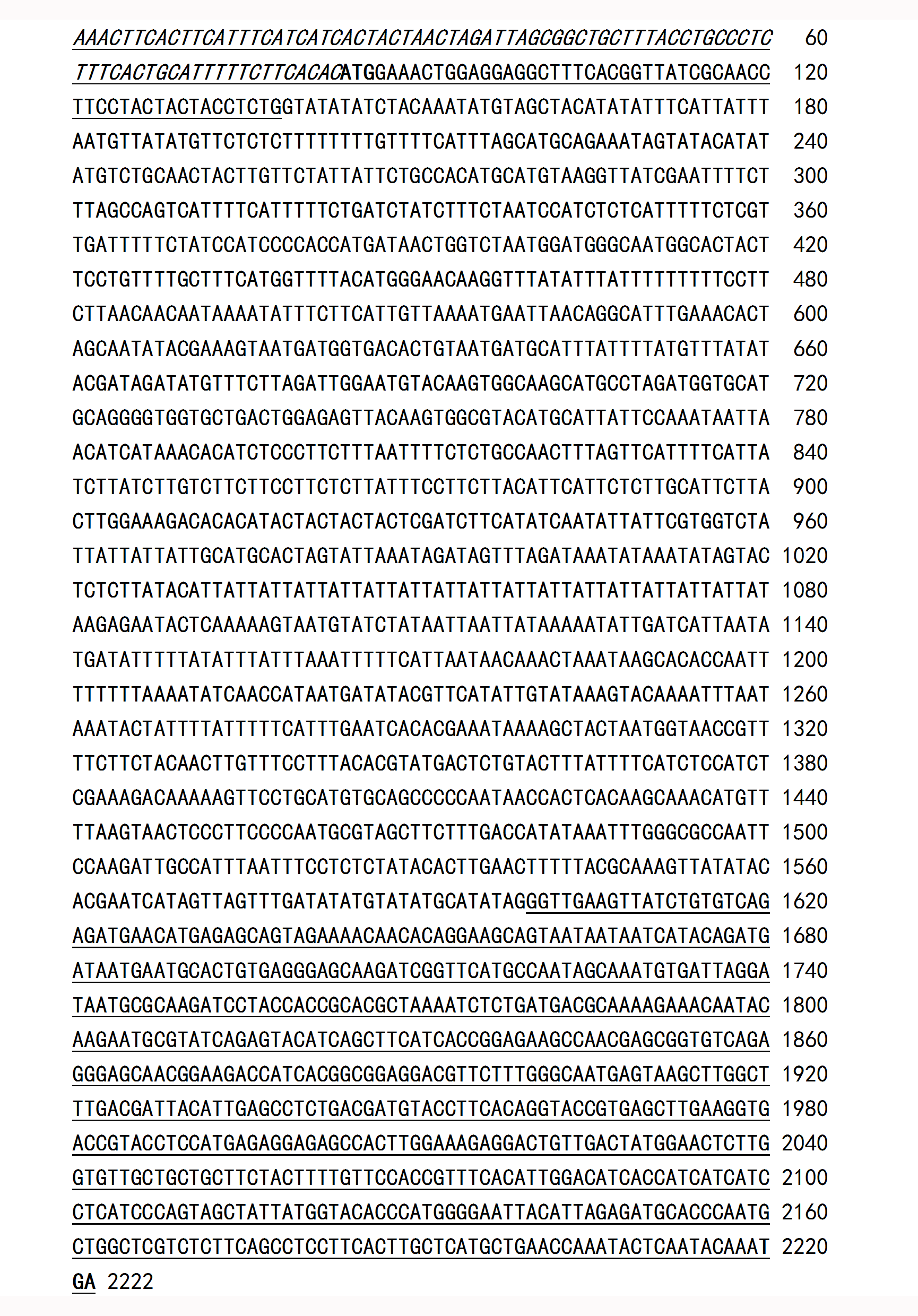

Supplement: S1 Fig — The sequences underlined indicated the exon1 and exon2, and the italics showed the 5′UTR sequence. The start and stop codon were shown using the bold letters. (TIF) [file pone.0139213.s001.tif]
